# Oligonucleotides-Based Therapeutics

**DOI:** 10.3390/biomedicines9101355

**Published:** 2021-09-29

**Authors:** Bernard Lebleu

**Affiliations:** Laboratory of Pathogen Host Interactions, Université Montpellier, UMR 5235 CNRS, 34000 Montpellier, France; marinaetbernard@lebleu.net

This Special Issue of *Biomedicines* aims to outline nucleic-acid-based strategies that have emerged as tools to regulate specific gene expression and, more recently, as a new class of medicines.

The possibility to down-regulate gene expression (namely, Rous Sarcoma virus) with short complementary (or antisense) chemically synthesized oligonucleotides was first established in a series of publications by the group of P.Zamecnik at the Worcester Foundation in the late 1970s [1,2]. The elegance and simplicity of this antisense concept rapidly drove a large interest from academic laboratories (including our own) and led to the emergence of several start-up companies, as reviewed here by Agrawal [3].

Antisense oligonucleotides (ASO) down-regulate their mRNA targets either stoichiometrically by steric hindrance or through the activation of the cellular RNAse H, thus leading to a more efficient enzymatic-like mechanism of mRNA degradation. Rapid progress in nucleic acid chemistry gave rise to many ASO analogs with improved pharmacological properties, including increased resistance to nucleases degradation, enhanced binding to the RNA target, improved bioavailability, or reduced toxicity. Mechanisms of action and chemical modifications of ASOs have been comprehensively reviewed by Gagliardi and Ashisawa [4] in this issue.

New applications have emerged that include the regulation of pre-mRNA splicing (using ASO analogs that do not activate RNAse H) or the down-regulation of micro RNAs.

ASOs can also be designed to interfere with the expression or activity of proteins specifically. As an example, the expression of the STAT3 transcription factor can be inhibited by short double-stranded DNA fragments named decoy ODNs, as described by Kim et al. [5] in this issue.

However, translating these promising data gathered from cell cultures or animal (mostly murine) models of human diseases remained disappointing for a long time, even leading to skepticism about the clinical interest of nucleic acids-based strategies. The main limitations have included poor delivery to ASO intracellular (cytoplasmic or nuclear) targets across cellular membranes or initially unexpected in vivo toxicity and immunogenicity. Most nucleic acids indeed elicit innate immune responses by binding to Pattern Recognition Receptors (Toll-like receptors or TLR in particular). Detailed studies have shown that both sequence motifs and chemical structures are responsible for these interactions. They can be avoided, as sought in most applications, to decrease off-target effects. Alternatively, ASOs can be designed as TLR agonists for applications in immunotherapy, as described by Agrawal [3].

The first FDA approval of an ASO-based drug (Formivirsen) was granted in 1998 to ISIS Pharmaceuticals (now Ionis Pharmaceuticals) for the treatment of ocular cytomegalovirus retinitis in severely immunocompromised patients (AIDS-infected patients in particular). Of broader application was Mipomersen, approved in 2013 for the treatment of homozygous familial hypercholesterolemia. ASO-mediated regulation of the complex pre-mRNA splicing machinery has also led to several interesting clinical applications with the 2016 FDA approval of ASO-based drugs to treat Duchenne muscular dystrophy (DMD) and spinal muscular atrophy (SMA).

Further studies aiming at a better understanding of the underlying splice switching mechanisms allowed optimization of the ASO design, which could ultimately be translated clinically—as illustrated here by Flynn et al. [6]—for the Survival Motor Neuron (SMN) gene, whose deficient expression is responsible for SMA.

Capitalizing on RNA interference pathways with small interfering RNAs (siRNAs) has also led to potent strategies to down-regulate specific gene expressions. Several such drugs have been approved to treat liver-associated human diseases, such as TTR-mediated amyloidosis or acute hepatic porphyrias, both developed by the Boston-based Alnylam company.

The registration of several nucleic-acid-based drugs in the US and Europe (as reviewed by Jani et al., [7]) is characteristic of a field that became mature after years of often unsuccessful research. Among the many reasons for this shift are the continued efforts by academic laboratories, now large biotechs (such as Ionis Pharmaceuticals or Alnylam Pharmaceuticals) and big pharmaceuticals (such as Novartis or Astra-Zeneca), to optimize ON-based drugs design, to study their pharmacokinetics and biodistribution in animal models or clinical trials, and, importantly, to understand in detail their mode of action and their cellular trafficking. This latter important issue has been the object of many studies using, as examples, association with cell-penetrating peptides or lipid nanoparticles (LNPs), as reviewed here by Boisguerin et al. [8] or Vlatkovic [9]. Capitalizing on the key role of hydrophobic interactions on the delivery issue, Bazaz et al. [10] have engineered a new family of orthogonally hydrocarbon-modified CPPs with good efficiency and tolerability, as demonstrated with in vitro and in vivo models.

Strategies to overcome the key issue of endosome entrapment after endocytosis has been described extensively by Juliano [11] with original proprietary tools.

Delivery to specific organs or cell types remains a major hurdle, with major successes achieved in the liver using LNP association or asialoglycoprotein conjugation. Many studies concentrate on extra-hepatic (central nervous system in particular) or topical (for example, ocular) delivery. Specific problems dealing with kidney delivery and potential clinical applications were illustrated here by Carton-Garcia et al. [12]. Along the same lines, Wijesinghe et al. [13] extensively reviewed oligonucleotides-based therapies to treat inflammatory joint diseases such as rheumatoid arthritis, synovial inflammation, or cartilage degeneration with an emphasis on delivery tools.

Recent developments have included manipulating the human genome using the CRISPR strategy, which allows the targeted inclusion of DNA sequences, thus revolutionizing gene therapy. This would deserve a complete issue and has therefore not been covered here.

Finally, mRNA has become a tool allowing the generation of proteins for protein replacement therapies or new vaccination strategies, as evidenced by the recent successes in SARS-COVID vaccination with approved mRNA drugs from BioNTech/Pfizer (see the contribution of Vlatkovic, [9]) and Moderna.

Although approved drugs concern eucaryotic cells and viruses, research aiming at targeting bacterial pathogens are in development given the increased number of antibiotic-resistant strains, as extensively reviewed by Jani et al. [7] or illustrated by Najid et al. [14] using peptide-conjugated PNAs (Peptide Nucleic Acids).

This fast-growing list of approved drugs has demonstrated the great potential of nucleic acids-based medicines that will complement low molecular weight drugs or protein-based drugs in treating various human diseases, including previously undruggable ones. Key issues encountered in their chemical synthesis, design, and delivery have found solutions, and initial successes will probably lead to rapid developments. As pointed out already, however, targeting extra-hepatic tissues and improving endosomal escape remain major roadblocks that are now extensively studied.

Lipid nanoparticles (LNPs) have been developed by Alnylam in partnership with Inex Pharmaceuticals, leading to Patisiran, the first FDA-approved siRNA drug. In addition, a slightly different lipid formulation has been engineered by BioNTech [9] for mRNA delivery that led to the highly successful SARS-CoV2 vaccine. A key to LNPs’ efficiency is the inclusion in the formulations of lipids that become positively charged in the acidic environment of endocytic vesicles, thus leading to LNP conformational changes and the cytoplasmic release of their nucleic acids content. Despite these important successes, which will hopefully allow the control of the COVID pandemic, improvements are still actively sought out, with an emphasis on tissue-specific delivery.

An alternative strategy has involved the chemical conjugation of ligands, as already largely documented for delivering siRNAs and antisense oligonucleotides. For example, GalNac conjugation targets the asialoglycoproteins receptors at the surface of hepatocytes, a strategy that has led to the approval of several drugs treating liver-associated human diseases. The conjugation of low molecular mass ligands, aptamers, or antibodies is being investigated to improve cellular uptake, ease endosomal release, or target specific tissues [15]. It will reasonably lead to new clinical developments for treating a broad range of diseases in the near future.
